# TEvarSim: A genome simulator for transposable element (TE) variants

**DOI:** 10.1371/journal.pcbi.1013933

**Published:** 2026-01-30

**Authors:** Jian Miao, Dawei Li

**Affiliations:** Department of Immunology and Molecular Microbiology, Graduate School of Biomedical Sciences, Texas Tech University Health Sciences Center, Lubbock, Texas, United States of America; Tsinghua University, CHINA

## Abstract

Transposable element (TE) variants, the presence or absence of TE sequences such as LINE-1, Alu, SVA, and endogenous retroviruses, are a major source of genomic diversity and play critical roles in human health, evolution, and disease. As interest in TE variants grows, developing related methods and tools for detection has become increasingly important. However, rigorous benchmarking of TE variant detection methods remains limited due to the lack of accurate and scalable TE variant simulation platforms and the absence of reliable ground truth data. Here, we developed TEvarSim, a novel TE variant simulator that generates TE-containing genomic data in multiple formats, including genomes, short- and long-read sequencing data, and VCF files. TEvarSim supports both random and real-world TE insertions and deletions, including variants derived from pangenome graphs. It can rapidly simulate hundreds to thousands of synthetic chromosomes or genomes and model natural variation at the haplotype, individual, and population levels, making it well suited for large-scale studies. In addition, TEvarSim can directly compare simulated VCF files with TEs reported by TE detection tools, streamlining the benchmarking of TE genotyping methods. TEvarSim provides an all-in-one toolkit for simulating, evaluating, and improving TE variant detection, advancing our ability to accurately study TEs in health and disease in various species.

## Author summary

TEvarSim is the first all-in-one toolkit that generates transposable element (TE) variant-carrying genomes, sequencing reads, and VCF files, and directly compares simulated and predicted TE variants, providing a comprehensive solution for benchmarking TE detection and genotyping. It is the first simulator to incorporate real TE sequences, extract TEs from pangenome graphs, and introduce natural sequence variation across genomes, supporting biologically relevant TE research. TEvarSim is also the first to directly and automatically simulate both TE insertions and deletions, providing a better understanding of the full spectrum of TE polymorphisms. TEvarSim can rapidly simulate thousands of individual synthetic genomes containing TE variants.

## Introduction

Transposable elements (TEs), including LINE-1 (L1), Alu, SVA, and endogenous retrovirus (ERV), account for approximately 44% of the human genome [1]. TE variants, also referred to as polymorphic TEs or mobile element variations, are defined by the presence or absence of TE sequences in the genome. This dichotomy occurs both within an individual’s haplotypes and across individuals and is a significant source of genetic variation affecting human health and disease [2], as well as phenotypic diversity in animals [3] and plants [4]. Driven by increasing interest in TE variant research, over 50 methods and tools for TE variant detection and genotyping have been developed in recent years [5–8]. However, their relative accuracies remain unclear, in part due to the lack of suitable computational models and benchmark tools for reliable evaluation. To help researchers select methods and software appropriate for specific study designs and data types, a comprehensive benchmarking framework is urgently needed, one that can accurately simulate TE-variant-containing synthetic genomes and sequencing datasets to rigorously evaluate tool performance.

Several tools have been developed to simulate TEs on reference genomes across different species, including SimulaTE [9], replicaTE [10], TEgenomeSimulator [11], and TE_simu [12]. However, except for SimulaTE, these tools were designed for benchmarking TE annotation. They generate a single simulated genome and do not support modeling TE variants across multiple samples. Consequently, they are not suitable for evaluating TE variant detection or genotyping. TE detection and simulation should include both TE insertion and deletion (i.e., the absence of a TE sequence relative to the reference genome). While SimulaTE can simulate TE insertions, simulating deletions requires users to manually create a TE-free reference genome to indirectly infer deletions, for example by excluding the presence of each specific TE. Another key limitation of SimulaTE is that it models TE insertion sites without introducing sequence variation into TE insertions across individuals. Using such simulated data for benchmarking may therefore favor tools that rely on known candidate TE variants for genotyping, such as GraffiTE [13], since the TE sequences in benchmarking samples are identical to those in the candidate TE variants. In real sequencing data, however, this advantage does not exist due to natural sequence diversity across individuals and populations. Therefore, introducing nucleotide variation into TE sequences among individuals is necessary for comparisons that better reflect real conditions.

Additionally, as long-read sequencing produces more complete genomes, an increasing number of pangenomes have been released. The graph-based pangenomes can capture real-world polymorphic TEs and incorporate population-wide structural variation. They are therefore an excellent data source for simulating the presence and absence of a broad spectrum of real TEs, including both polymorphic insertions and deletions. Based on these considerations, developing new simulators is essential for reliable benchmarking and method development. We therefore developed TEvarSim, a new tool designed to enable more accurate, comprehensive, and scalable simulation of TE variants for various species.

## Design and implementation

### Software design

**Fig 1:**
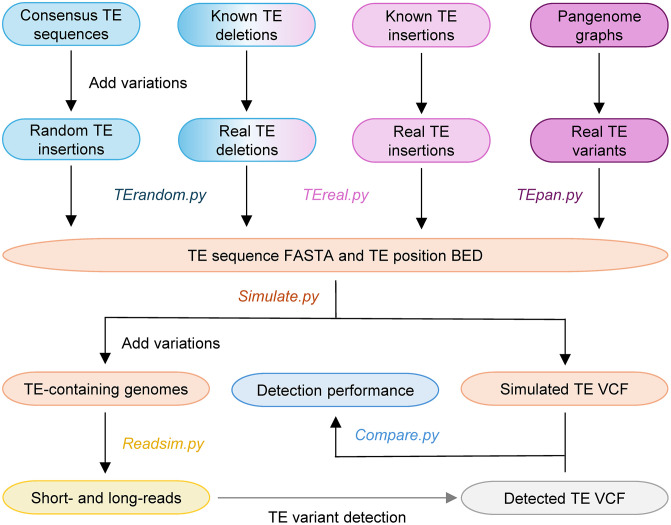
Software workflow of TEvarSim. TEvarSim consists of four Python modules: First module (*TErandom.py*, *TEreal.py*, and *TEpan.py*): Creates TE insertion sequences and BED files containing the coordinates of TE insertions and deletions. Known TE deletions are used by both *TErandom.py* and *TEreal.py*. Users must select one and only one of the three parallel methods (*TErandom*, *TEreal*, or *TEpan*) per simulation run. Second module (*Simulate.py*): Generates TE variant-containing genomes and corresponding VCF files based on the simulated TE variants. Third module (*Readsim.py*): Produces short- and long-read sequencing data from the simulated genomes. Fourth module (*Compare.py*): Compares the simulated VCF files with files from TE variant genotyping tools to evaluate detection accuracy.

The workflow of TEvarSim consists of four modules: TE variant generation, TE-harboring synthetic genome simulation (*Simulate*), read simulation (*Readsim*), and TE variant comparison and benchmarking (*Compare*). TEvarSim provides three parallel methods for TE variant generation: random (*TErandom*), real (*TEreal*), or from pangenome (*TEpan*), with users selecting just one method per simulation run. During read simulation, TEvarSim can generate both short- and long-read data from the synthetic genomes. An overview of the TEvarSim workflow is shown in **Fig 1**.

### Create random TE variants

The first module’s random TE method, *TErandom*, is used to generate TE variants purely at random. It requires two input files: (i) TE consensus sequences (or user-defined custom sequences) and (ii) reference genome coordinates of known TE variants for simulating insertions and deletions, respectively. *TErandom* introduces sequence variation into TE consensus sequences by randomly generating single nucleotide polymorphisms (SNPs), insertions/deletions (INDELs), 3’ polyA tails, and 5’ truncations, based on user-defined parameters (see **Software Manual**). These variations are embedded in the insertion sequences and annotated in their sequence identifiers. The resulting sequences are used as simulated TE insertions, with their positions randomly assigned across the reference genome. Presence or absence of each insertion in different samples is also randomly assigned. In parallel, the start and end positions of TE deletions are derived from the coordinates of known TE variants in the reference genome (or user-defined custom variants). The specific TEs to delete are randomly selected from these known variant sites. Finally, *TErandom* merges the simulated TE insertions and deletions and outputs a BED file containing their genomic locations and a FASTA file containing the sequences of all simulated TE insertions.

### Create TE variants from real samples

The first module’s real TE method, *TEreal*, is used to simulate the presence and absence of TE variants based on TE variants observed in real genomes. Unlike *TErandom*, this *TEreal* method does not use consensus sequences or randomly generate insertion sites. Instead, it requires (i) a file of known TE insertions and (ii) a FASTA file containing their sequences. Sequence identifiers must follow the RepeatMasker [14] format: chromosome-position-sequence description (e.g., chr1-683234-AluSp#SINE/Alu), where the TE superfamily name is required for functions that perform stratified analyses by TE superfamily. To simplify the procedure, we have provided a pre-curated dataset of 168,600 high-quality real TE insertion sequences for direct use, which were recently identified from 130 near-complete human genomes [15]. TE deletion simulation in *TEreal* follows the same procedure as in *TErandom*.

### Create TE variants directly from pangenome graphs

The first module’s pangenome method, *TEpan*, automatically identifies TE variants, including both insertions and deletions, directly from real sample pangenome graphs. Since pangenome graphs contain real structural variation, *TEpan* extracts TEs from these variable regions of the graphs. *TEpan* takes a Graphical Fragment Assembly file as input and identifies structural variants using gfatools [16]. It then employs RepeatMasker to annotate repeats and extract sequences matching user-specified TE families. The sequences and genomic positions of the identified TE variants are extracted from the pangenome graphs, generating corresponding FASTA and BED files. The three parallel methods in the first module, *TErandom*, *TEreal*, and *TEpan*, operate independently, with only one applied per simulation run. To generate data using all three methods, each must be executed separately.

### Simulate genomes and generate VCF files

The second module, *Simulate*, generates synthetic genomes containing TE variants along with corresponding VCF files. For each TE variant, presence or absence is randomly assigned based on user-defined parameters such as allele frequency. Using the FASTA and BED files produced by *TErandom*, *TEreal*, or *TEpan*, this module outputs the variant-containing genomes and a corresponding VCF file. In SimulaTE, genome simulation requires processing all TE insertions for each simulated genome by cutting and merging sequences, resulting in time complexity *O*(m·n·c), where n is the number of TE insertions, m is the number of simulated genomes, and c is genome length. To improve efficiency, TEvarSim employs a chunk-merge strategy, splitting each genome into blocks based on TE variant breakpoints specified in the BED file. These blocks are classified as either variable (containing TE variants) or invariable (unaltered reference sequence). The presence of each variable block is determined by the genotypes recorded in the VCF file. When simulating multiple genomes, these blocks are concatenated according to the presence or absence of each TE block. This strategy significantly accelerates simulation of multiple genomes, reducing time complexity to *O*(m·(n+c)). Optionally, users can enable a parameter to introduce sequence variation (e.g., SNPs and INDELs) within each TE variant across genomes, allowing simulation of natural sequence diversity. However, enabling this feature increases computational time, as each insertion sequence must then be individually modified for each genome.

### Simulate short- and long-read sequencing data

The third module, *Readsim*, generates short- and long-read sequencing data from the simulated genomes. TEvarSim randomly cuts each genome (or chromosome) into reads using Mason2 [17] for short-read simulation and PBSIM3 [18] for long-read simulation. Reads are generated for each simulated genome according to user-defined parameters. For Illumina short reads, users can configure parameters such as sequencing depth, read length, average fragment length, and standard deviation of fragment length. For PacBio and Nanopore long reads, parameters include sequencing depth, base error rate, average read length, and standard deviation of read length.

### Compare simulated and predicted VCF files

The fourth module, *Compare*, evaluates concordance between a simulated VCF and a VCF or BED file containing predicted TEs generated by a third-party TE variant genotyping or detection tool. Existing TE simulation tools typically do not produce VCF files or provide functionality to assess detection accuracy. This module addresses that gap. It takes two input files: the simulated VCF (serving as ground truth) and the predicted TEs from the tool being evaluated. Since some tools cannot output TEs in VCF format, the predicted TE file can be provided in either VCF or BED format. The module first assesses positional concordance by matching simulated and predicted coordinates within a user-defined positional mismatch tolerance. It then calculates and reports standard performance metrics, including true positives, false positives, false negatives, sensitivity (recall), precision, and F1-score. For TE variants that are positionally matched, the module further evaluates genotype concordance and reports genotyping accuracy. Additionally, it outputs the matched variants and their genotypes into a user-specified file, allowing users to directly compare and access all evaluated results. Since some tools only detect TE insertions without genotyping them, the *Compare* module also supports only evaluating the presence or absence of TE insertions without assessing genotypes.

## Results

We verified that the genomes simulated by TEvarSim are consistent with those produced by SimulaTE, confirmed that the variants recorded in the VCF file were correctly incorporated into the simulated genomes, and demonstrated that sequence variation was successfully introduced. We also provide a proof-of-concept workflow illustrating the use of TEvarSim to benchmark real TE detection tools on both short- and long-read datasets. The following paragraphs summarize the findings from these evaluations.

### Comparison with other tools

We summarized the key features of four existing TE simulation tools in **Table 1**, including SimulaTE, replicaTE, TEgenomeSimulator, and TE_simu. Except for SimulaTE, the other three tools were designed specifically for benchmarking TE annotation from assembled genomes. Thus, they are limited to generating a single genome per run and do not capture natural TE sequence diversity. These tools cannot model the presence or absence of TE sequences across individuals, making them unsuitable for TE variant detection or genotyping studies. Even with SimulaTE, unless users provide a TE-free reference genome, TE deletions cannot be automatically simulated. However, preparing such a TE-free reference genome requires custom scripting, which may introduce a barrier for biologists without programming experience. Moreover, using a TE-free reference disrupts the coordinate system of the standard reference genome, making it difficult in practice to simulate real-world TE variants with known reference genomic coordinates. Given these limitations, we focused our direct comparison on SimulaTE without simulating TE deletions.

**Table 1:** Comparison of five TE simulation tools.

	TEgenome Simulator	TE_simu	replicaTE	SimulaTE	TEvarSim
TE insertion simulation	Y	Y	Y	Y	Y
TE deletion simulation*	–	–	–	–	Y
TE variant simulation	–	–	–	Y	Y
Consensus TE variation	Y	Y	Y	Y	Y
Real TE inclusion	–	–	–	–	Y
TEs on pangenomes	–	–	–	–	Y
Genome variation*	–	–	–	–	Y
One genome simulation	Y	Y	Y	Y	Y
Mult-genome simulation	–	–	–	Y	Y
VCF output	–	–	–	–	Y
Reads simulation	–	–	–	Y	Y
Detection tool evaluation	–	–	–	–	Y

* Direct and automated simulations with no need for manual effort by users.

**Fig 2:**
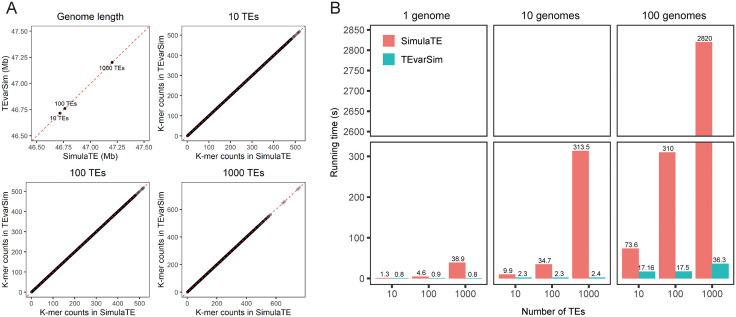
Comparison with an existing tool. **(A)** Comparison of TE-containing genomes simulated by SimulaTE and TEvarSim, showing identical outputs. **(B)** Runtime comparison between SimulaTE and TEvarSim using a single CPU and thread.

We downloaded human TE consensus sequences from Dfam [19] and used SimulaTE to insert 10, 100, and 1,000 TE sequences into chromosome 21 of the human reference genome GRCh38 (GRCh38-Chr21). We selected chromosome 21, the shortest human chromosome, as a proxy for whole-genome simulation. To match SimulaTE’s simulation settings, we used an in-house script to generate a BED file recording the same simulated events, which was then used for downstream TEvarSim simulations. First, we verified that the genome lengths simulated by TEvarSim exactly matched those generated by SimulaTE. Using Jellyfish [20], we then extracted k-mers from each simulated genome and confirmed that the counts of each unique k-mer were identical. These results demonstrate that the genomes produced by both tools are identical, supporting the accuracy of TEvarSim’s genome simulation (**Fig 2A**). To evaluate runtime performance, we simulated 1, 10, and 100 genomes using each tool. Under the same computational resources (a single CPU and thread), TEvarSim showed a clear runtime advantage, particularly when simulating larger numbers of genomes (**Fig 2B**). This improvement is attributable to our new simulation strategy, which avoids redundant genomic sequence processing. Together, these results indicate that TEvarSim is especially well suited for large-scale TE genome and pangenome simulations.

### Consistency between simulated genome and VCF

**Fig 3:**
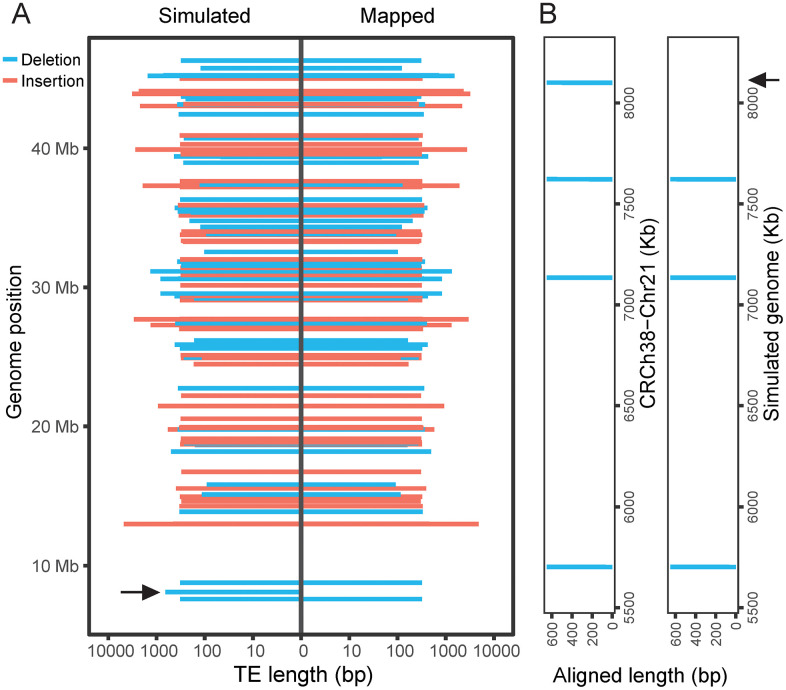
Validation of simulated genomes. **(A)** Comparison of TE variants recorded in the VCF file (left) versus those extracted from CIGAR strings in the BAM file after minimap2 alignment (right). A discrepancy in one TE deletion is highlighted with an arrow. **(B)** When the sequence of the TE deletion in question was mapped to both GRCh38-Chr21 and the simulated genome, it aligned fully to GRCh38-Chr21 but not to the expected location in the simulated genome, indicating that the deletion was successfully introduced. This sequence had four multi-mapped positions on the reference and three on the simulated genome, likely contributing to the minimap2 mapping artifact.

To validate consistency between the simulated genome and the corresponding VCF file generated by TEvarSim, we used the *TEreal* module to simulate a genome with 50 TE insertions and 50 TE deletions based on GRCh38-Chr21. We aligned the simulated genome to GRCh38-Chr21 using minimap2 [21], then extracted TE variant information from the CIGAR strings using an in-house script and compared it with the entries in the VCF file. All TE variants listed in the VCF were confirmed by the minimap2 alignment, with one exception: a TE deletion at chr21:8,100,087-8,100,744 (**Fig 3A**). We extracted this deletion sequence from GRCh38-Chr21 and re-mapped it to both the reference and simulated genomes. The sequence mapped to the reference but not the simulated genome, indicating that the TE deletion was correctly simulated. Furthermore, the sequence also mapped to three additional locations (**Fig 3B**), suggesting that the discrepancy was due to a minimap2 mapping artifact rather than a simulation error.

### Validation of simulated sequence variation

**Fig 4:**
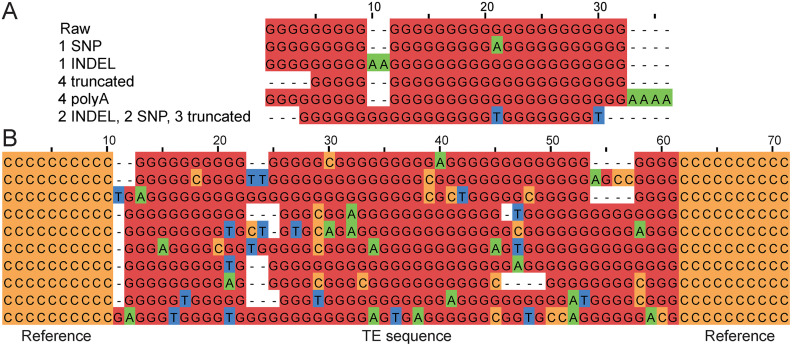
Validation of sequence variation. **(A)** Multiple sequence alignment showing that random sequence variations, including SNPs, INDELs, polyA tails, and truncations, were correctly introduced into TE consensus sequences. **(B)** Multiple sequence alignment demonstrating the expected sequence diversity of the same TE insertion across different individual genomes.

We validated TEvarSim’s function to simulate sequence variation using multiple sequence alignments. First, we confirmed that SNPs, INDELs, polyA tails, and truncations introduced into TE consensus sequences were correctly embedded (**Fig 4A**). Second, insertions of the same TE across different simulated genomes (individuals) exhibited the expected sequence diversity (**Fig 4B**). These results confirm that TEvarSim reliably generates sequence polymorphisms at both the TE and genome levels.

### Examples of TE detection tool benchmarking workflow using TEvarSim

As a benchmarking platform, TEvarSim requires concrete validation through systematic evaluation of existing TE detection tools by comparing their performance on simulated datasets with known ground truth. We used two TE detection tools, MELT [5] for short reads and xTea [22] for long reads, as proof-of-concept examples to demonstrate the overall workflow and practical utility of TEvarSim for benchmarking TE detection or genotyping tools. First, we used the *TErandom* module to simulate 2,200 TE variants across the 22 autosomes of GRCh38. The human TE consensus sequences included nine Alu sequences and two SVA sequences from the Dfam database, as well as one L1 sequence and one ERV-K sequence from MELT itself. Using the *Simulate* module, we generated a synthetic genome for subsequent analyses. Simulation frequencies of TE variants were randomly sampled between 0.1 and 0.9, resulting in a synthetic human genome containing 439 Alu, 42 L1, 73 SVA, and 55 ERV-K insertions. Subsequently, 30 × short reads and long reads were generated using the *Readsim* module and analyzed for TE variant detection with MELT and xTea, respectively. Their performance was then assessed using the *Compare* module.

**Fig 5:**
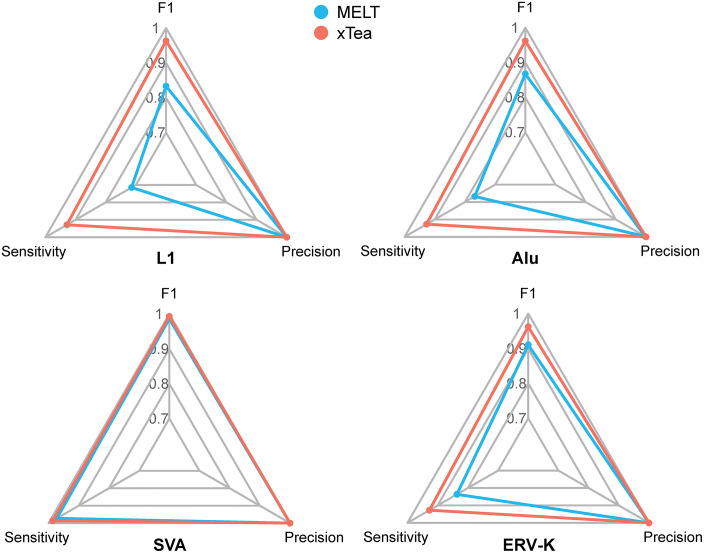
Radar charts of performance metrics of MELT and xTea using TEvarSim-simulated TE-containing short-read and long-read datasets, respectively.

For TE variant detection, the results showed that, based on short reads, the F1 scores of MELT were 0.83, 0.87, 0.99, and 0.91 for L1, Alu, SVA, and ERV-K, respectively, whereas, based on long reads, xTea achieved higher F1 scores of 0.96, 0.96, 0.99, and 0.96, respectively (**Fig 5**). For TE variant genotyping, the genotyping accuracy of MELT (defined as the percentage of TE variants with exactly identical genotypes among all correctly matched TE variants from TE detection) was 1, 0.73, 0.38, and 0.85 for L1, Alu, SVA, and ERV-K, respectively. Genotyping was not evaluated for xTea because it does not generate genotypes when using long reads. **Table 2** shows the detection sensitivity, precision, and F1 scores, as well as genotyping accuracy across different TE types. The analysis code for the proof-of-concept benchmarking workflow, from genome simulation to performance evaluation, is included in the TEvarSim software. We recognize that this proof-of-concept demonstration remains limited in breadth (e.g., a limited number of tools and sequencing conditions) and is restricted to a basic comparison of two randomly selected tools. Consequently, findings from this example should not be interpreted as comprehensive. Nevertheless, this workflow demonstrates the feasibility of TE detection benchmarking. With this proof-of-concept framework, more systematic and comprehensive benchmarking analyses can be readily extended to more advanced and complex scenarios by modifying the workflow pipeline, including stratified analyses [23–25] across different sequencing depths and genomic contexts, as well as applications to a wide range of existing TE variant detection or genotyping tools.

**Table 2:** Performance metrics of two randomly selected TE detection tools.

Tools	TE type	True number	Predicted number	TE detection			Genotyping
				Sensitivity	Precision	F1 score	Accuracy*
MELT	L1	42	30	0.71	1	0.83	1
	Alu	439	338	0.77	0.997	0.87	0.73
	SVA	73	71	0.97	1	0.99	0.38
	ERV-K	55	46	0.84	1	0.91	0.85
xTea	L1	42	39	0.93	1	0.96	–
	Alu	439	407	0.93	1	0.96	–
	SVA	73	72	0.99	1	0.99	–
	ERV-K	55	51	0.93	1	0.96	–

* Genotyping accuracy is defined as the percentage of TE variants with exactly identical genotypes among all correctly matched TE variants from TE detection.

-, xTea provides genotypes only for short-read data, not long-read data.

Performance was evaluated using simulated datasets with 30 × sequencing depth based on all 22 pairs of autosomal chromosomes of the human reference genome GRCh38.

Additionally, we evaluated how key simulation parameters in the *TErandom* modules influence output quality and benchmarking results of existing TE detection tools. First, we identified key parameters that may affect the variation of synthetic genomes, focusing on five parameters related to genomic variation simulation (**S1 Table**). We then assessed how varying degrees of genomic variation in TE sequences, implemented by adjusting these parameters, affect benchmarking performance, using MELT as an example. We compared the default parameters with a set introducing smaller variation and a set introducing larger variation. To quantify variation in the simulated genomes, we calculated the genome Mash distance [26] between each simulated genome and the reference genome. We found that all simulated genomes exhibited very small Mash distances relative to the reference genome and that, as expected, the Mash distance increased as the level of variation introduced to TEs increased (**Table 3**). No significant performance changes were observed between the default and lower-variation parameter sets. However, as expected, higher variation in TE consensus sequences significantly decreased the power of the TE detection tool. For example, the average TE detection F1 score across all TE types decreased from 0.81 (default parameters) to 0.52 (higher-variation parameters) for MELT. **Table 3** shows the changes in TE detection sensitivity, precision, F1 score, and genotyping accuracy for MELT. The overall results aligned with expectations, reflecting the current challenge that existing tools still face in detecting TEs substantially divergent from consensus sequences. This further emphasizes the urgent need to develop new algorithms capable of accurately identifying real-world TE variants not represented by consensus sequences. We acknowledge that the current comparisons are limited to five parameters related to genomic variation simulation and to a single randomly selected tool, and that additional parameters and TE detection tools should be evaluated systematically in future studies. The performance of TE detection is largely determined by the design of each tool, and different tools may perform differently under varying simulation conditions. The *TErandom* module provides users with flexibility in defining simulation conditions tailored to specific needs, although selecting appropriate parameters can sometimes be challenging for new users. Due to the diverse use cases for TE simulation, it is also difficult to objectively estimate how key parameters (e.g., insertion rate, variant length, and mutation rate) influence simulation outputs. In practice, however, users can obtain a comprehensive view of simulated TE variants by examining the BED files generated by TEvarSim. For beginners who may still struggle with selecting suitable parameters for the *TErandom* module, the *TEreal* module can be used as an alternative, as it simulates the presence and absence of known TE insertions. Overall, based on our current findings, we anticipate that TEvarSim has strong potential to support benchmarking of existing TE detection tools as well as the development of new methods.

**Table 3:** Comparison of MELT performance across genomic variation parameter sets in TEvarSim simulations.

TEvarSim parameter sets	Mash distance	TE type	True number	Predicted number	TE detection			Genotyping
					Sensitivity	Precision	F1 score	Accuracy
Default	4.8 × 10^-5^	L1	4	4	1	1	1	1
		Alu	45	34	0.76	1	0.86	0.88
		SVA	7	6	0.86	1	0.92	0.33
		ERV-K	4	5	1	0.8	0.89	1
		Average	60	59	0.8	0.81	0.81	0.83
Low variation	0	L1	1	1	1	1	1	1
		Alu	48	38	0.79	1	0.88	0.84
		SVA	6	5	0.67	0.8	0.73	1
		ERV-K	5	6	1	0.83	0.91	1
		Average	60	60	0.8	0.8	0.8	0.88
High variation	2.4 × 10^-5^	L1	4	0	0	0	0	0
		Alu	35	8	0.23	1	0.37	0.88
		SVA	9	4	0.44	1	0.62	0
		ERV-K	12	9	0.75	1	0.86	0.11
		Average	60	60	0.35	1	0.52	0.38

The five key parameters for simulating genomic variation in TE consensus sequences include: 1) SNP mutation rate per base, 2) INDEL mutation rate per base, 3) geometric distribution parameter for INDEL lengths, 4) proportion of sequences to truncate, and 5) maximum proportion of sequences to truncate. The “default” values for these parameters are 0.02, 0.005, 0.7, 0.3, and 0.5, respectively. The “lower variation” and “higher variation” values are 0.01, 0.001, 0.8, 0.2, and 0.4; and 0.05, 0.01, 0.5, 0.4, and 0.6, respectively (**S1 Table**). Performance was evaluated using simulated datasets with 10 × sequencing depth based on chromosome 1 of the human reference genome GRCh38. Average performance was calculated by combining all TE types.

## Availability and future directions

### Software availability

TEvarSim is implemented in Python 3. All source code, scripts, and data, including those for the proof-of-concept benchmarking workflow, are publicly available at https://github.com/daweili-lab/TEvarSim. Additional materials can be found at www.dllab.org/software/TEvarSim.html.

### Documentation

TEvarSim provides documentation at three levels: (i) interactive command-line help messages built into the software, providing a preview of the usage of each parameter; (ii) GitHub repository containing step-by-step usage instructions, recommended practices, example datasets, and other resources to support testing and reproducibility; and (iii) **Software Manual** available on our laboratory webpage with additional project information, Q&A, and contact details.

### Computational resource requirements

The memory requirements of TEvarSim scale with chromosome length, e.g., simulating a human chromosome typically requires less than 8 GB of RAM. Disk usage varies depending on factors such as the number of genomes simulated, the size of each genome (e.g., partial or whole), and the sequencing depth of the simulated reads. **Table 4** provides an example summary of resource consumption for simulations based on chromosome 1 of GRCh38.

**Table 4:** Example computational resource consumption.

No. of genomes	Disk usage (GB)	Peak memory usage (GB)	CPU hours
10	2.4	3.79	0.014
100	24	3.79	0.048
1,000	236	3.8	0.44

Simulations were performed on chromosome 1 of the human reference genome GRCh38 using a single CPU and single thread.

### Limitations and future directions

The current version of TEvarSim has several limitations that motivate future development. Specifically, the simulation modules based on random (*TErandom*) and real (*TEreal* and *TEpan*) TEs are complementary to each other, with each having distinct strengths and limitations. Overall, the random TE module can include TE sites and genomic regions not currently detectable by existing bioinformatics tools and is therefore more suitable for simulating understudied TE families for which little prior knowledge is available. An obvious limitation of this approach is that it does not necessarily incorporate prior biological knowledge. In contrast, the real TE modules better reflect known insertion patterns observed in real samples and are better suited for scenarios in which TEs have been relatively well characterized, with most TE variants identified and a database of known TE variants available. Ideally, real TE sequences should be directly used as references to simulate the presence and absence of real TE insertions, as implemented in our current real TE modules. Theoretically, these real TE modules can generate a more realistic TE landscape. However, current TE detection methods remain in their early stages, and only a fraction of TE variants have been identified in the literature and in our in-house TE variant database. In particular, TEs located in highly repetitive regions have not been accurately identified or characterized. Consequently, the currently available human TE variants and their sequences are far from complete, and this limitation is even more severe for many other species that lack comprehensive databases of real TE insertions. Thus, it is still necessary to include a random TE module to generate randomized TE variants, supporting the study of TEs in both easily identifiable and challenging genomic regions.

Under its default settings, the random module in our current software version does not preferentially increase or decrease the prevalence of specific TE variant types in particular genomic regions. However, depending on the specific biological question, users may wish to enrich for or against TE variants in specific regions, such as complex TE insertions in highly repetitive regions. This functionality can be readily implemented. TEvarSim uses BED files produced in prior steps to generate synthetic genomes through the *Simulate* module. Therefore, inserting, enriching, or excluding TE variants in specific genomic regions (e.g., repetitive regions) can be achieved by modifying the BED file before running the *Simulate* module.

We also acknowledge that our current simulation framework applies uniform parameters across all TE types when inserting TEs and introducing sequence variations. In the real world, different TE types and families likely have distinct insertion preferences (e.g., Alu elements may favor GC-rich regions, whereas L1 elements prefer AT-rich regions), as well as different structural characteristics and mutation patterns in the TE sequences and their flanking regions. Future versions could include a new module that incorporates the strengths of both the random and real TE modules by implementing TE type- and family-specific simulation parameters. For example, repeat annotations from the reference genome could be parsed to characterize family-specific TE variants and their variation patterns, generating differentiated simulation parameters for TE insertion locations and sequence variation across TE families. This new module should capture well-established biological differences among TE types, as well as other known family-specific features, such as divergence patterns and evolutionary constraints. In addition, although TEvarSim currently does not support multi-CPU execution, parallel execution may be implemented in future versions to further improve simulation speed.

## Conclusion

TEvarSim is an all-in-one, easy-to-use toolkit for simulating TE variants. It can simulate genomes containing random or real TE insertions and deletions, including those derived from pangenome graphs. The toolkit generates both short- and long-read sequencing data and can produce tens of thousands of synthetic genomic samples incorporating real-world individual- and population-level genetic variation. To address the recognized need for benchmarking TE detection methods, TEvarSim also generates and compares VCF or BED files for comprehensive evaluation and improvement of TE variant detection and genotyping in a controllable and reproducible environment. Because TE variant and genome information are stored in editable BED and FASTA files, users can easily fine-tune simulations (e.g., enriching or excluding TEs in specific regions) or extend TEvarSim to model other types of genomic variation (e.g., SNPs, INDELs, structural variants, or specific sequence elements) and adapt simulations to other species. TEvarSim is readily applicable to TE detection, genotyping, and related research in humans, animals, and plants.

## Supporting information

S1 Table Key parameters for simulating genomic variation in TE consensus sequences. (PDF)
