# Supplementary material for: TEvarSim: A genome simulator for transposable element (TE) variants
Source: PLoS Comput Biol. 2026 Jan 30;22(1):e1013933. doi: 10.1371/journal.pcbi.1013933 (PMC12875575; doi:10.1371/journal.pcbi.1013933)
Supplement: S1 Table — (PDF) [file pcbi.1013933.s001.pdf]

Supporting Information

S1 Table Key parameters for simulating genomic variation in TE consensus sequences

Key parameter	Description	Default value	Lower variation	Higher variation
snp-rate	SNP mutation rate per base	0.02	0.01	0.05
indel-rate	INDEL mutation rate per base	0.005	0.001	0.01
indel-geom-p	Geometric distribution parameter for INDEL lengths	0.7	0.8	0.5
truncated-ratio	Proportion of sequences to truncate	0.3	0.2	0.4
truncated-max-length	Maximum proportion of sequences to truncate	0.5	0.4	0.6
